# *Drosophila* Homeodomain-Interacting Protein Kinase (Hipk) Phosphorylates the Hippo/Warts Signalling Effector Yorkie

**DOI:** 10.3390/ijms22041862

**Published:** 2021-02-13

**Authors:** Eva Louise Steinmetz, Denise Nicole Dewald, Uwe Walldorf

**Affiliations:** 1 Developmental Biology, ZHMB (Center of Human and Molecular Biology), Saarland University, Building 61, D-66421 Homburg, Germany; eva.steinmetz@uni-saarland.de (E.L.S.); denise.dewald@uni-tuebingen.de (D.N.D.); 2 Department 8.3 Biosciences, Zoology/Physiology-Neurobiology, ZHMB (Center of Human and Molecular Biology), Saarland University, Building B2.1, D-66123 Saarbrücken, Germany; 3 Inter-Faculty Institute for Cell Biology Animal Genetics, Auf der Morgenstelle 15, D-72076 Tübingen, Germany

**Keywords:** homeodomain-interacting protein kinase (Hipk), serine/threonine kinase, Hippo/Warts signalling, Yorkie (Yki)

## Abstract

Developmental growth and patterning are regulated by an interconnected signalling network of several pathways. In *Drosophila*, the Warts (Wts) kinase, a component of the Hippo signalling pathway, plays an essential role in regulating transcription and growth by phosphorylating its substrate Yorkie (Yki). The phosphorylation of Yki critically influences its localisation and activity as a transcriptional coactivator. In this study, we identified the homeodomain-interacting protein kinase (Hipk) as another kinase that phosphorylates Yki and mapped several sites of Yki phosphorylated by Hipk, using in vitro analysis: Ser168, Ser169/Ser172 and Ser255. These sites might provide auxiliary input for Yki regulation in vivo, as transgenic flies with mutations in these show prominent phenotypes; Hipk, therefore, represents an additional upstream regulator of Yki that works in concert with Wts.

## 1. Introduction

The regulation of growth is an especially important developmental process in all organisms. Organs derive their final shapes and sizes from a fine balance between cell proliferation and apoptosis. In genetic screens in the model organism *Drosophila*, four tumour-suppressor genes—*warts* (*wts*), *salvador* (*sav*), *hippo* (*hpo*) and *mob as a tumour suppressor* (*mats*)—were discovered to regulate organ growth. Their mutations in cell clones lead to massive overgrowth phenotypes, and they all belong to the same signalling pathway, the Hippo pathway, which is evolutionarily conserved up to mammals (reviewed in [1]). The core of the pathway is currently described as a canonical kinase cascade of tumour suppressors, notably, the kinase Hippo (Hpo) in complex with Salvador (Sav) (MST1/2-SAV1 complex in mammals), which phosphorylates and activates a complex of the kinase Warts (Wts) and Mob as tumour-suppressor (Mats) (the LATS1/2-MOB1A/B complex in mammals), which, in turn, phosphorylates and inactivates its direct and physiological substrate Yorkie (Yki) (YAP/TAZ in mammals). The identification of the non-DNA-binding transcriptional coactivator Yki resolved the missing link between Wts and transcriptional regulation. In the absence of Hpo signalling, unphosphorylated Yki enters the nucleus and forms a nuclear complex with Scalloped (Sd) (TEAD in mammals), a DNA-binding transcription factor in combination with which it regulates the expression of Hpo target genes, resulting in an increase in cell number and tissue size [2,3]. Any Yki/YAP-induced alteration in the expression of Hpo target genes is genetically dependent on endogenous Sd/TEAD. A default repression model has been proposed in which in the absence of Yki, Sd engages transcriptional corepressors that actively repress the transcription of Hpo pathway target genes [2,4,5].

In *Drosophila* as well as in mammals, the phosphorylation of Ser168(Yki)/Ser127(YAP) by Wts/LATS1 critically impacts the outcome of the Hpo signalling pathway, as it inactivates the potential oncogene by excluding it from the nucleus through an interaction with the protein 14-3-3 [6,7,8,9,10,11,12,13,14]. Further phosphorylation sites (Ser111, Ser169, Ser172 and Ser250) were found to contribute to the regulation of Yki in flies, as mutations of these sites reduce the phosphorylation of Ser168 [12,13,14]. Ser111 and Ser250 were shown to be targets of Wts, while the kinase(s) responsible for the phosphorylation of Ser169 and Ser172 have not yet been described. In mammals, YAP activity might be influenced by additional LATS1 phosphorylation sites (Ser61, Ser109, Ser164 and Ser381), which were identified according to the LATS1 consensus motif HXRXXS [9,15,16].

Further studies have found additional kinases converging on the Hpo signalling cascade in mammals, such as CDK1, AMPK, SRC, NLK, IKKε and PRP4K (reviewed in [2]). In *Drosophila*, the homeodomain-interacting protein kinase (Hipk) was delineated as promoting Yki’s transcriptional activity [17,18]. The serine/threonine protein kinase family of HIPKs/Hipk is conserved throughout the animal kingdom [19,20]. In flies, only one member of the HIPK family is known as an ortholog, compared to the four representatives within vertebrates [21,22]. The protein domain structures of the best-investigated and thus prototypical human HIPK2 and the *Drosophila* Hipk show a high degree of homology, which is as high as 83% for the catalytic kinase domain [23]. They play roles in a variety of developmental and pathological processes. These predominantly nuclear-occurring kinases regulate gene transcription pathways, proliferation, cell survival, differentiation and the response to DNA damage [24,25,26]. Several studies have revealed a link between Hipk and the regulation of growth, in part, by stimulating Notch signalling through interference with the formation of corepressor complexes [18,27,28]. In the context of Hippo signalling, Chen and Verheyen showed that Hipk promotes Yki-mediated growth and target gene expression without affecting Yki’s stability or subcellular localisation, by promoting its transcriptional activity within the nucleus [17]. They also stated that Yki interacts via its WW domain with the PPxY motifs of many Hpo pathway components, including Wts. As Hipk also possesses a conserved PPxY sequence, its interaction with Yki might be mediated in the same way [17,29]. Furthermore, they showed that the overgrowth phenotype and target gene expression caused by hyperactive Yki are suppressed by *hipk* loss-of-function mutations [17]. Another group identified Hipk as a component of the Hippo signalling pathway during an RNA interference screen [18], in parallel with the work of Chen and Verheyen. They showed that Hipk is involved in, but not essential for, basal Yki activity and that it is likely to regulate Yki’s function by promoting its accumulation in the nucleus, thereby affecting Yki/YAP activity by acting downstream of or in parallel with Wts/LATS1 and LATS2 [18]. In both studies, Hipk was described as the first kinase identified to positively regulate Yki [17,18]. In this study, we analysed the direct enzyme–substrate relationship of Hipk and Yki in detail. We mapped several sites at which Yki can be phosphorylated through in vitro analysis and subsequently tested the newly identified sites that Hipk can phosphorylate by the targeted misexpression of correspondingly mutated Yki variants with destroyed Hipk-phosphorylation sites. We thereby obtained the first evidence for the functional relevance of the identified phosphorylation sites in vivo.

## 2. Results

### 2.1. Hipk Phosphorylates Yorkie at Several Sites

In *Drosophila*, Yorkie (Yki) is regulated by Warts-mediated phosphorylation at Ser111, Ser168 and Ser250 and, furthermore, was supposed to be a substrate of yet-to-be-identified protein kinase(s) acting on the residues Ser169 and Ser172 [7,8,12,13]. As Hipk had already been shown to phosphorylate Yki [17], our intention was to identify the phosphorylation sites of Yki that might be specific Hipk targets. 

**Figure 1:**
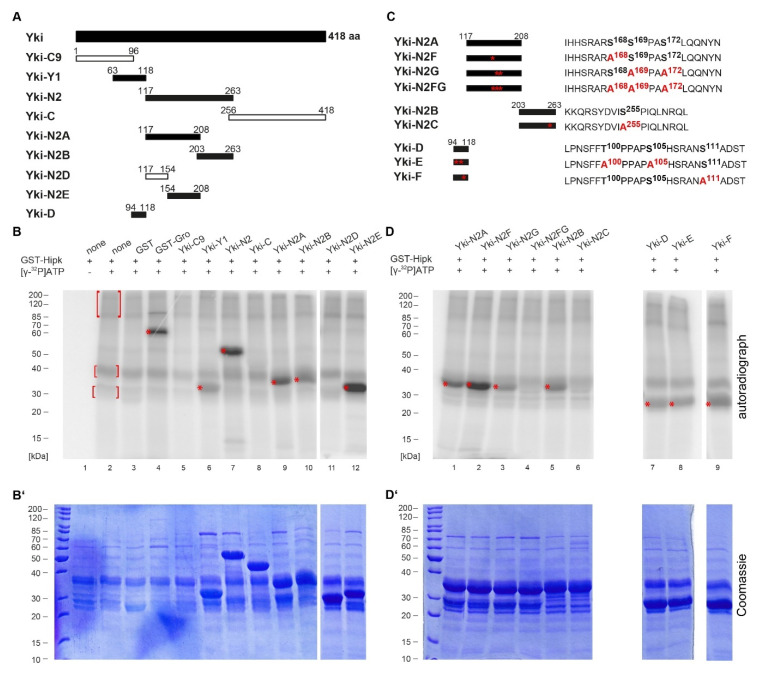
Homeodomain-interacting protein kinase (Hipk) phosphorylates Yorkie (Yki) in vitro. (**A**) Schematic view of the full-length protein Yki with 418 amino acids (aa). Positions and sizes of different constructs of Yki that were purified and analysed by phosphorylation assay are shown below (Yki-C9, -Y1, -N2, -C, -N2A, -N2B, -N2D, -N2E and -D). Numbers indicate terminal amino acid positions of the constructs. Phosphorylated constructs are represented as black and non-phosphorylated constructs as white bars. (**B**) Autoradiograph after in vitro phosphorylation assay of the purified Yki constructs. Each protein contained a glutathione S-transferase (GST)-tag and was incubated with full-length GST-Hipk and radioactively labelled adenosine triphosphate ([γ-^32^P]ATP). GST-Hipk and GST protein alone served as negative controls (lanes 2 and 3), and GST-Groucho (Gro), a confirmed substrate of Hipk, as a positive control (lane 4). Autophosphorylation of Hipk led to a background signal (see marked within red brackets), which was subtracted in the following lanes. Signals from phosphorylated proteins are marked with red asterisks. (**B’**) Loading control according to (B): original 12%-SDS (sodium dodecyl sulfate)-polyacrylamide gel electrophoresis (PAGE) for the phosphorylation assay, Coomassie-stained. (**C**) Schematic view of the mutated derivatives of Yki-N2A (Yki-N2F, -N2G and -N2FG), Yki-N2B (Yki-N2C) and Yki-D (Yki-E and Yki-F) with a section of the amino acid sequence of the corresponding fragment on the right. Potentially phosphorylated amino acid residues that were affected by the mutational analysis are accentuated in bold; the respective mutated sites are shown in red. (**D**) Autoradiograph after in vitro phosphorylation assay of the mutated Yki constructs. Signals from phosphorylated proteins are marked with red asterisks. (**D’**) Loading control according to (D): original 12%-SDS-PAGE for the phosphorylation assay, Coomassie-stained.

For better handling, we initially divided the protein sequence of Yki into smaller sections; each was amplified from the protein-coding region of *yorkie* and cloned for the expression of recombinant proteins. The resulting GST (glutathione S-transferase) fusion proteins were affinity-purified and subjected to an in vitro phosphorylation assay with recombinant GST-Hipk and radiolabelled [γ-^32^P]ATP. A schematic overview of all the Yki fragments tested is shown in Figure 1A. For an initial rough mapping, Yki was divided into an N-terminal fragment (Yki-C9, aa 1–96), two middle fragments (Yki-Y1, aa 63–118, and Yki-N2, aa 117–263) and a C-terminal fragment (Yki-C, aa 256–418). The results of the corresponding in vitro phosphorylation assay are shown in Figure 1B (loading control in 1B’). The N- and C-terminal parts, Yki-C9 (lane 5) and Yki-C (lane 8), were not phosphorylated by Hipk, while there were at least two target regions in the middle part of Yki: Yki-Y1 (lane 6) and Yki-N2 (lane 7). As the construct Yki-C9 showed no phosphorylation, we could narrow down the phosphorylated region to amino acid residues 97–118. This was confirmed by generating another N-terminal subconstruct, Yki-D (Figure 1A), which was positively tested for phosphorylation (Figure 1D, lane 7). Next, we divided Yki-N2 into the subconstructs Yki-N2A (aa 117–208) and Yki-N2B (aa 203–263), both showing phosphorylation signals. Yki-N2B’s signal was weaker than that of Yki-N2A but still clearly detectable (lanes 9 and 10). To further analyse fragment Yki-N2A, it was subdivided into Yki-N2D (aa 117–154) and Yki-N2E (aa 154–208), which narrowed down the phosphorylated region to Yki-N2E because this subconstruct was phosphorylated while Yki-N2D was not (lanes 11 and 12); therefore, we were able to locate phosphorylation to three distinct regions of Yki: aa 97–118, aa 154–208 and aa 203–263.

We performed a mutational analysis of the three identified regions to identify the specific amino acid residues (serine or threonine) phosphorylated by Hipk; we introduced single amino acid changes from serine or threonine to alanine. As Hipk has been described as a proline-guided serine/threonine kinase with phosphorylation target sites that mostly appear in clusters of two or three adjacent residues around a proline according to the consensus S/X-R/X-S/T-P-S/X [30,31,32], we focused on these most promising positions within the Yki sequence; however, we also tested some potentially phosphorylatable serine and threonine residues, which could have represented less obvious Hipk sites, as they did not match our earlier defined consensus [31], nor were they in the vicinity of prolines [30]. We generated completely mutated versions of the subconstructs in which all the potential phosphorylation sites were changed to alanine, and constructs with single potential phosphorylation sites mutated to check them separately. For the mutational analysis of Yki-N2A, we generated Yki-N2F, Yki-N2G and Yki-N2FG (Figure 1C) and tested them under equal conditions. The in vitro phosphorylation assay implied that Ser168, Ser169 and/or Ser172 are the only Hipk phosphorylation sites in this subconstruct (aa 117–208) because the simultaneous mutation of these sites led to a total loss of the phosphorylation signal (see Figure 1D, lane 4). The mutation of Ser168 or Ser169/Ser172 alone, respectively, did not eliminate the phosphorylation signal completely (Figure 1D, lanes 2 and 3), which implies that not only Ser168 but also Ser169/Ser172 are phosphorylated by Hipk. The phosphorylation site Ser168 matches the Wts-phosphorylation site described by others [8,12,13], which raises the possibility that this site might be a shared target of Wts and Hipk. In fact, the surrounding amino acid positions match both the already described substrate consensus for LATS1 (mammalian homologue of Wts) and our Hipk substrate consensus [9,31].

Although Ser169 and Ser172 have been excluded as Wts-phosphorylation sites by some researchers, they have been examined for their influence on Ser168 phosphorylation [13]. When both sites were mutated to alanine, the N-terminal Yki seemed to become a better substrate for phosphorylation by the endogenous kinases of S2 cells, and when phosphomimetic mutations were introduced at Ser169 and Ser172, the full-length Yki seemed to be less phosphorylated [13]. In our in vitro assay, when Ser168 was mutated to alanine (Yki-N2F), Yki was more strongly phosphorylated by Hipk than the wildtype form Yki-N2A (compare Figure 1D, lanes 1 and 2). The stronger phosphorylation implies that the mutation of Ser168 to alanine promotes the Hipk-mediated phosphorylation at Ser169/172, which might be due to some conformational changes induced by the physicochemical properties of the phosphate group. Conversely, when Ser169/Ser172 were simultaneously mutated to alanines, Yki was still phosphorylated by Hipk (Yki-N2G; lane 3) but less strongly than wildtype Yki-N2A. When all three sites (Ser168, Ser169 and Ser172) were mutated, the phosphorylation signal was no longer present (Yki-N2FG; lane 4). Therefore, Hipk phosphorylates both Ser168 and Ser169/Ser172, and that of the latter is even enhanced when Ser168 is mutated. In vivo, this fits with the observation that Yki bearing a mutated Ser168 is not phosphorylated by Wts and therefore not bound to 14-3-3 [8,12]. The consequence of not being retained in the cytoplasm by 14-3-3 is that Yki translocates into the nucleus [8,12] and becomes accessible for phosphorylation by nuclear Hipk.

Another Hipk-phosphorylation site was also suggested to exist within Yki-N2B; this fragment was positively tested in the above-mentioned assay. We decided to focus on Ser255 as a potential Hipk target based on our consensus. The mutation of Ser255 to alanine (Yki-N2C; Figure 1C) completely eliminated the phosphorylation signal that was originally observed in wildtype Yki-N2B (compare Figure 1D, lanes 5 and 6), clearly confirming Ser255 as a Hipk-phosphorylation site.

Our studies described thus far of the Yki-C9 fragment, multiply-mutated Yki-N2FG fragment and Yki-N2C fragment showed that, at least in vitro, there are no Hipk phosphorylation sites at the N-terminal end of Yki (aa 1–96) and no further Hipk phosphorylation sites within the C-terminal region of Yorkie (aa 117–418). 

To complement the analysis of the N-terminal region of Yki, we generated mutated versions of Yki-D (aa 94–118, Figure 1A). Thr100 and Ser105 approximately matched our Hipk consensus [31,32] and therefore became the focus of our analysis. We also examined another position, Ser111, which had already been described as a Wts-phosphorylation site in Yki [9,15], although there was no agreement with the Hipk consensus (see Figure 1C,D, lanes 7, 8 and 9). We mutated Thr100 and Ser105 simultaneously (Yki-E) or Ser111 alone (Yki-F), considering them as potential Hipk-phosphorylation sites. Neither mutation could eliminate the slight phosphorylation signal, leading us to assume that the observed phosphorylation site was not affected and that the signal came from a phosphorylation of either Ser97, Ser107, Ser114 or Thr115—the only remaining phosphorylatable amino acid residues in this construct (Figure 1C,D, lanes 7, 8 and 9). However, none of those residues is located in the direct vicinity of a proline, usually needed for recognition by Hipk, and therefore we did not pursue any further investigation of this sequence area and focused on the functional analysis of the clearly mapped phosphorylation sites. Not every phosphorylation site identified in vitro is necessarily phosphorylated in vivo, as it could be masked due to the 3D folding of the protein under physiological conditions. However, all of our mapped residues were identified as being phosphorylated by unknown kinases in a general phosphoproteomic analysis of *Drosophila* embryos [33], and thus they should generally also be accessible for Hipk in vivo and were, therefore, considered worthy of further examination.

**Figure 2:**
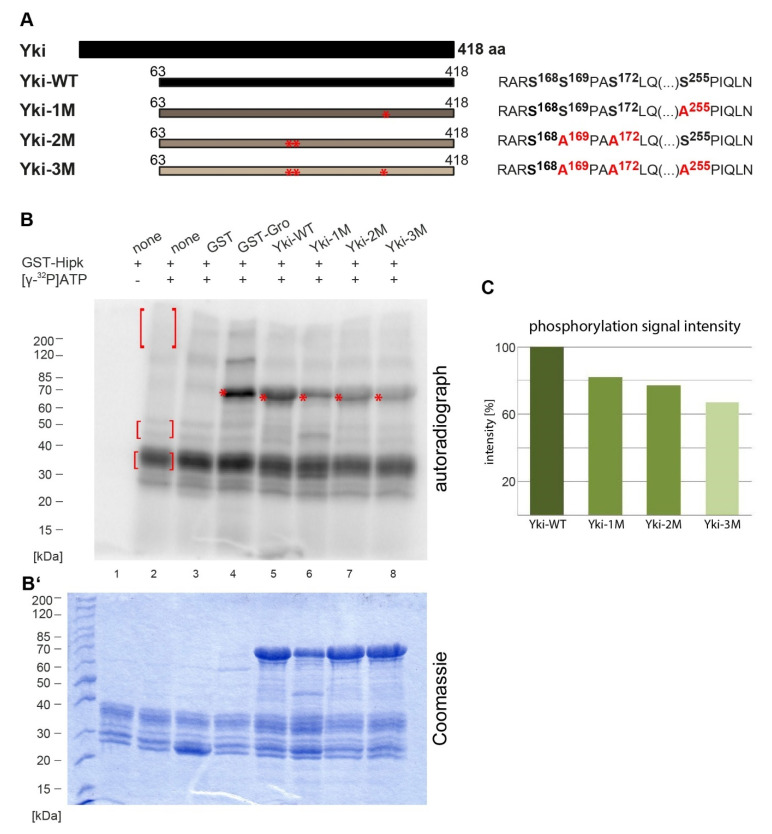
(**A**) Schematic view of Yki with 418 amino acids (aa). Positions and sizes of different constructs of Yki that were purified and analysed by phosphorylation assay are shown below (Yki-WT, -1M, -2M and -3M). Red asterisks highlight mutated amino acid positions; the respective amino acid sequences are shown alongside. Amino acid residues affected by the mutations are accentuated in bold; the respective mutated sites are shown in red. (**B**) Autoradiograph after in vitro phosphorylation assay of the purified Yki constructs. Each protein contained a GST-tag and was incubated with full-length GST-Hipk and radioactively labelled adenosine triphosphate ([γ-^32^P]ATP). Controls were performed as described in Figure 1. Autophosphorylation of Hipk led to a signal (see marked within red brackets), which was subtracted from substrate phosphorylation signals in the following lanes. Signals from phosphorylated proteins are marked with red asterisks. (**B’**) Loading control according to (**B**): original 12%-SDS-PAGE from the phosphorylation assay, Coomassie-stained. Equal amounts of protein were used in the assay (for Yki-WT, -1M, -2M and -3M). (**C**) Quantification of the phosphorylation signal intensities according to B, using ImageJ.

Following the previous analysis using small Yorkie fragments, we aimed to perform a comparative in vitro phosphorylation assay with the full-length wildtype protein and its mutated forms, to verify if the proposed sites acted as major Hipk phosphorylation sites. Due to the properties in the amino-terminal area (aa 1–63) of Yorkie, the protein could not be expressed and purified efficiently, but an N-terminally truncated form (aa 63–418) worked well and was used; it is referred to as the wildtype form of Yki (Yki-WT) for the following experiments. The tested GST fusion proteins are shown in Figure 2. Yki-WT was strongly phosphorylated (Figure 2B, lane 5), while the mutated forms (Yki-1M, Yki-2M and Yki-3M) were weakly phosphorylated (Figure 2B, lanes 6, 7 and 8). The proteins were tested in equal amounts (using the original SDS-PAGE shown in Figure 2B’ as a loading control) for comparability. Yki-1M produced an additional band, highlighting a tendency towards degradation (Figure 2B, lane 6). In Yki-3M, all the phosphorylation sites described above as Hipk targets (Ser169, Ser172 and Ser255) were mutated; this showed the weakest phosphorylation signal. We also checked Yki-4M for phosphorylation by Hipk, in which all the above-stated Hipk targets and Ser168 were mutated. This form showed a phosphorylation signal almost equal to that of Yki-3M (see Supplementary Material, Figure S1), probably because the size of the analysed fragment and, thus, the resulting 3D folding can influence the accessibility of the sites and, thus, the strength of the cumulated phosphorylation signal.

### 2.2. Hipk Phosphorylation Regulates Yorkie In Vivo

**Figure 3:**
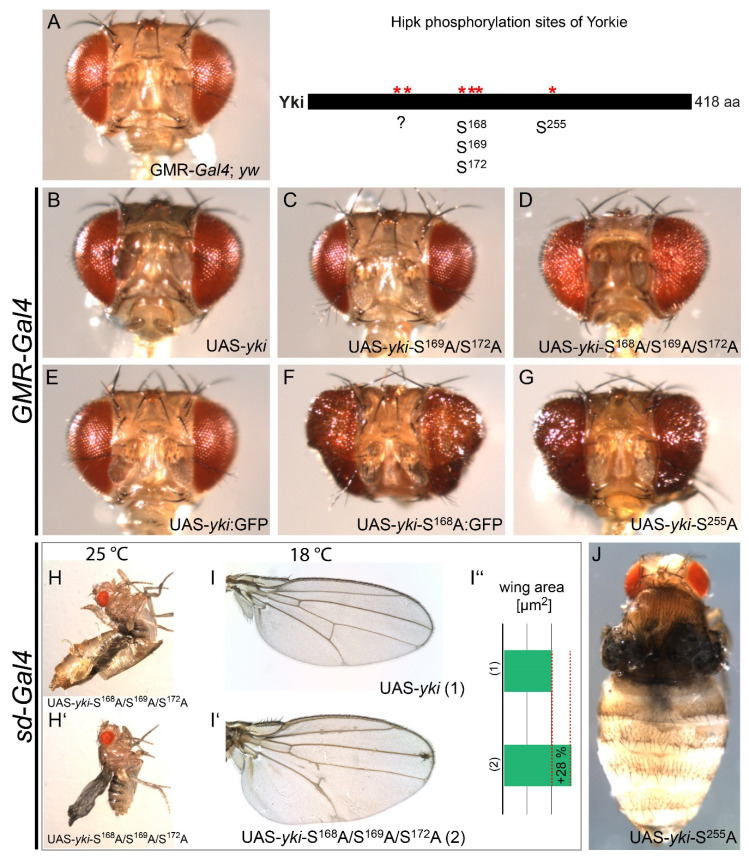
Phosphorylation at Ser169/Ser172 and Ser255 contribute to the Yki activity in vivo. (**A–G**) Heads of adult female flies with the *glass multiple reporter* (*GMR*)-*Gal4* and (**A**) no UAS (upstream activation sequence) transgene with a background of *yellow white* (*yw*^67c23^) (BL 6599), (**B**) UAS-*yki*, (**C**) UAS-*yki-*S^169^A/S^172^A, (**D**) UAS-*yki-*S^168^A/S^169^A/S^172^A, (**E**) UAS-*yki*:GFP (BL 28815; Oh and Irvine, 2008), (**F**) UAS-*yki*-S^168^A:GFP (BL 28816; Oh and Irvine, 2008) and (**G**) UAS-*yki-*S^255^A. (**H–J**) Adult female flies with *scalloped* (*sd*)-*Gal4* and (**H**,**H’**) UAS-*yki-*S^168^A/S^169^A/S^172^A (25 °C), (**I**) UAS-*yki* (18 °C), and (**I’**) UAS-*yki-*S^168^A/S^169^A/S^172^A (18 °C); (**I’’**) wing area measurements according to I and I’: (1) UAS-*yki* (female wings, *n* = 10) with an average area of 2,084,600 µm^2^, and (2) UAS-*yki-*S^168^A/S^169^A/S^172^A (female wings, *n* = 10) with an average area of 2,913,488 µm^2^; (**J**) adult female fly with *scalloped* (*sd*)-*Gal4* and UAS-*yki-*S^255^A.

The identification of several Hipk phosphorylation sites in Yki (Ser168, Ser169/Ser172 and Ser255) led us to the question of whether Yki might be regulated in vivo by these posttranslational modifications. To answer this, appropriate transgenic *Drosophila* strains were generated by site-directed integration at the same chromosomal position, to avoid any effects of the insertion point on expression levels and ensure comparability. Each transgene bore a modified UAS (upstream activation sequence)-*yki* construct according to an identified Hipk phosphorylation site: UAS-*yki-*S^169^A/S^172^A, UAS-*yki-*S^168^A/S^169^A/S^172^A and UAS-*yki-*S^255^A. We also generated a UAS-*yki* strain without mutations as an internal reference; this strain was also compared to the already described UAS-*yki*:GFP (Figure 3B,E; [12]) expressed under the same conditions as a benchmark. The expression of the UAS transgenes under the control of the *glass multiple reporter* (*GMR*)-*Gal4* (eye-specific) as well as *scalloped* (*sd*)-*Gal4* (wing-specific) was examined (Figure 3). As homozygous flies of the driver line *GMR*-*Gal4* exhibit a slight rough eye phenotype, we first assessed flies heterozygous for the *GMR*-*Gal4* transgene, using them as the control phenotype (Figure 3A). The expression of UAS-*yki* led to a slightly overgrown eye phenotype (Figure 3B). This may be due to endogenous tumour suppressors, which can keep even such an increased level of Yki in check so long as the major Wts-phosphorylation site at Ser168 is accessible. The eyes from flies after the misexpression of UAS-*yki*-S^169^A/S^172^A appeared marginally smaller (Figure 3C) than eyes after the misexpression of UAS-*yki*. A comparable phenotype was found by Oh and Irvine [13], who studied the influence of mutated Ser169 and Ser172 on Ser168 without knowing the corresponding kinase. Their work further supports our observations, as phosphomimetic mutations led to reduced phosphorylation at Ser168 and decreased affinity for 14-3-3. By contrast, the destruction of both sites led to increased phosphorylation at Ser168, an increased affinity for 14-3-3 [13] and, therefore, decreased Yki activity, which could account for the slight reduction in eye size. The destruction of Ser168 alone led to an evident overgrown eye phenotype due to the loss of Wts phosphorylation and, therefore, an increased nuclear localisation and activation of Yki in both studies (Figure 3F; [12,13]).

The expression of the triple mutant UAS-*yki-*S^168^A/S^169^A/S^172^A in our assay led to a rough but only minor overgrown eye phenotype (Figure 3D). The observed phenotypic differences led us to assume that the disruption of the phosphorylation sites Ser169/Ser172 lowers the activity of Yki. As we showed in our in vitro phosphorylation assay that the affected residues are Hipk-phosphorylation targets, this milder phenotypical effect might be due to attenuated Hipk signalling. This appears to be supported by the observation of Oh and Irvine that a mutation of Ser169/Ser172 to alanine leads to increased phosphorylation at Ser168 [13], as the phosphorylated protein analysed in their work might represent not only Yki protein phosphorylated by Wts but also the nuclear fraction of Yki modified by Hipk.

The eye-specific expression of UAS-*yki-*S^255^A led to a dramatically overgrown eye phenotype (Figure 3G), implying great Yki activation. To our knowledge, Ser255 is described here for the first time as an important site for the regulation of Yki, and our phosphorylation data allow us to ascribe this regulatory activity, at least partly, to Hipk.

When expressing the UAS-*yki* transgenes specifically in the wing using *sd*-*Gal4*, the strength of the resulting phenotypes is similar to the results in the eye. When UAS-*yki-*S^168^A/S^169^A/S^172^A was expressed at 25 °C, the flies were apparently unable to hatch completely from their pupal cases, presumably due to enlarged wings that stuck to the pupal cases (Figure 3H,H’). To investigate this further, we repeated the crosses at a lower temperature (18 °C) and could rescue the lethal phenotype: the expression of UAS-*yki* did not lead to a significant phenotype (Figure 3I), while the expression of UAS-*yki-*S^168^A/S^169^A/S^172^A led to visibly enlarged wings (Figure 3I’). Based on this, we measured the area of the wing after the induced expression of UAS-*yki-*S^168^A/S^169^A/S^172^A in comparison to that of a wing after the expression of UAS-*yki* (Figure 3I”; synchronously raised at 18 °C). The wing area was enlarged by 28% (UAS-*yki*: 2,084,600 µm^2^, on average, compared to UAS-*yki*-S^168^A/S^169^A/S^172^A: 2,913,488 µm^2^, on average; female wings, *n* = 10).

Expressing UAS-*yki-*S^255^A via *sd-Gal4* also led to a lethal phenotype that could not be rescued at lower temperatures. The wings of the flies extracted manually from the pupae were malformed, and we assume they were not everted correctly during metamorphosis; this probably led to death inside the pupal case (Figure 3J).

## 3. Discussion

The Hippo signalling pathway represents a major control mechanism for developmental tissue growth, and its functional dysregulation has enormous oncogenic potential; therefore, the regulation of this pathway and, especially, its key regulator Yorkie (Yki) has been of great scientific interest in recent years (reviewed in [2,3]). In 2012, two groups independently identified Hipk as a positive regulator of Yki activity but came to divergent conclusions about the actual mechanism of regulation [17,18]. While Poon et al. found Hipk to affect the localisation of Yki but no direct evidence for an enzyme–substrate relationship, Chen and Verheyen demonstrated the phosphorylation of Yki by the kinase. Our study now confirms the work of Chen and Verheyen, as we were able to map the Hipk-mediated phosphorylation of Yki in vitro to several distinct residues (Ser168, Ser169/Ser172 and Ser255) and a further target region (aa 94–118, Figure 1; Yki-D) that has not been fully clarified. Even after the mutation of the sites that came into question according to the Hipk consensus and the preference for a proline-guided position [30,31], this region was still phosphorylated by Hipk in vitro; however, we set aside a further analysis of this region and focused on the role of the identifiable residues. As the latter better agreed with the consensus and, furthermore, the respective constructs mostly yielded stronger phosphorylation signals, we assumed that those residues might also play more important functional roles.

The close-together sites Ser168 and Ser169/Ser172 are of special interest, as they are all already known to be phosphorylated [12,13]. Ser168 is a substrate for the Hippo pathway core component Wts [12]. A single residue being phosphorylatable by two kinases might illustrate an interesting form of differential regulation. Wts is located in the cytoplasm, where its phosphorylation of Ser168 promotes the binding of Yki to 14-3-3 proteins, retaining Yki in the cytoplasm [12]. Hipk, on the other hand, is known to be a nuclear kinase [19] and, thus, can influence the activity of Yki on a different, subcellular level. In this case, the two kinases would not compete for the target site, as only the Yki protein with non-phosphorylated Ser168 should be imported into the nucleus. The other sites, Ser169 and Ser172, have also been shown to be phosphorylated, although not by Wts but by a kinase unknown at the time of analysis. Phosphorylation at both sites reduced the phosphorylation at Ser168 and promoted the activity of Yki [13]. As we have shown that Hipk can phosphorylate all three sites, it is possible that the phosphorylation at Ser168 detected by Oh and Irvine represents not only the fraction modified by Wts but also a fraction targeted by Hipk, whose phosphorylation at Ser168 is influenced by the phosphorylation states of the adjacent residues. The described enhancement of Yki activity, if Ser169 and Ser172 are phosphorylated, and the identification of those sites as Hipk targets in our study are also in accordance with Hipk acting as a positive regulator of Yki [17]. The in vivo effect of the mutation of these two phosphorylation sites on the size of the wings has already been analysed by another group [13] and appeared similarly weak as the effect we observed in the eye, in contrast to a phosphomimetic mutation, which led to overproliferation effects in the wings [13]. Since the manipulation of the phosphorylation sites was independent of any kinase that could be responsible for it in vivo, it is entirely conceivable that the observed overproliferation could also be the result of activation of Yki by Hipk.

These sites (Ser168, Ser169 and Ser172) are well conserved in human YAP [9,15], and thus phosphorylation by Hipk might be a common mechanism for the fine control of Yki/YAP activity and tissue growth. The phosphorylation of S168 by Wts is also conserved [7,8], and therefore the respective serine residue might serve as a target for differential regulation in various organisms.

As members of the Hipk family play various and sometimes even key roles during development and the Hippo signalling pathway represents an important switch for the regulation of proliferation and overgrowth, linking these Hipk sites could provide further insight into the connection between the (de)regulation of proliferation events in the physiological as well as in the pathological sense.

In contrast to the other mapped sites, Ser255 is not associated with Wts/LATS phosphorylation in vertebrates [9,15]; however, considering the evolutionary distance between flies and vertebrates, it seems highly probable that the different evolutionary paths also developed additional, specific mechanisms for finetuning growth regulation. To our knowledge, this newly identified Hipk phosphorylation site has not been mentioned elsewhere; however, it matches data from a phosphoproteomic analysis of *Drosophila* embryos that established Ser255 as a site of Yki that is phosphorylated in vivo by an unknown kinase [33]. The same analysis revealed some more sites in Yki phosphorylated in vivo during embryonic development: Ser105, Ser111, Ser114, Thr115, Tyr116, Ser120, Ser122, Ser123, Ser168, Ser169, Ser172, Ser250, Tyr251 and Ser255 [33]. These comprise, amongst others, the already known Wts/LATS-phosphorylation sites Ser111, Ser168 and Ser250 [9,15] and the phosphorylation sites Ser169 and Ser172, which do not represent Wts/LATS-phosphorylation sites [13] but were able to be identified as Hipk targets in this work. 

Ser255 is registered as a high-potential possible target site of the extracellular signal-regulated kinase (ERK; consensus motif: V-X-**S**/T-P) in the database of phosphorylation sites, PHOSIDA [34]. ERK kinases belong to the group of mitogen-activated protein kinases (MAPK) that exhibit extremely broad substrate specificity, including that for both nuclear and cytoplasmic proteins [35]. Other kinases additionally phosphorylate numerous Hipk/HIPK2 substrates, and the proline-guided MAPKs deserve particular mention. For example, HIPK2 and MAP kinase p38 phosphorylate the transcription factors PDX-1 (Ser269, [36,37]) and p53 (Ser46, [38,39]) at the same phosphorylation site; therefore, Ser255 might also participate in cooperative regulation through several kinases.

In the above-mentioned phosphoproteomic screen, Tyr251 in Yki was identified as an in vivo target of phosphorylation by an unknown kinase [33], and Ser250 in Yki has already been described as a Wts-phosphorylation site [9,13,15]. Both are located in the direct vicinity of Ser255, which is phosphorylated by Hipk. Nevertheless, the results of our in vitro phosphorylation assay exclude the possibility that Ser250 is phosphorylated by Hipk, as the phosphorylation signal was absent after the mutation of the amino acid Ser255 to alanine (Figure 1D, lane 6), and therefore there must be at least one further kinase involved in Yki phosphorylation. Considering the results from our in vitro phosphorylation analysis of the Yki region aa 96–118, which show potential Hipk-phosphorylation sites at Ser97, Ser107, Ser114 and Thr115, a phosphorylation at Ser114 or Thr115 seems the most likely despite the lack of a guiding proline, as the data from the in vivo phosphoproteomic screen also match these two sites [33]. It will be interesting to analyse if there is a mechanism of cooperative regulation involving all these phosphorylation sites in further studies.

To assess the functional relevance of the newly identified Hipk phosphorylation sites, we analysed the ectopic expression of several phosphomutants of Yki where one or more of the mapped target sites were replaced by a non-phosphorylatable alanine. The expression of UAS-*yki*-S^168^A via *GMR-Gal4* leads to a significant overgrowth phenotype as described previously due to the lack of phosphorylation by Wts and, therefore, increased nuclear localisation and activity of Yki [12]. The additional mutation of the Hipk-specific residues 169 and 172 weakens the overgrowth phenotype, implicating a loss of Hipk’s activating effect on Yki. These results are in line with an observation by Chen and Verheyen [17], where *hipk* knockdown rescued the overgrowth phenotype of Yki S168A. Although our triple mutant Yki-S168A/S169A/S172A does not fully prevent phosphorylation by Hipk as the knockdown does, Ser169 and Ser172 seem to mediate most of Hipk’s effect on Yorkie activity. This functional relationship and the evolutionary conservation of the respective residues argue, once again, for a potential conservation of a mechanism for the Hipk-mediated control of Yki.

Interestingly, the loss of phosphorylation at Ser255 leads to a strong overgrowth effect that seems to contradict the activating role of Hipk; however, as Hipk knockdown itself does not lead to an increase in eye size [28,31], it seems that the effect of Ser169 and Ser172 not being phosphorylated is sufficient to suppress the activating effect of non-phosphorylated Ser255. It cannot be excluded that Hipk itself exerts differential effects on different sites of Yki; that these effects are, to some extent, context-specific; or that further cofactors are involved in the nuclear regulation of Yki.

Phosphorylation at Ser255 could affect the interaction of Yki with various proteins, especially those that interact with Yki via WW1 (e.g., Wts, Ex, Hpo and NcoA6) [1,13,40]. Ser255 is not actually located in either of the two WW domains of Yki (WW1: aa 264–294; WW2: aa 333–363) but is remarkably close. Interactions via WW domains are dual and may have a positive or negative impact on the activity of Yki; therefore, Hipk phosphorylation at Ser255 might affect the repression mechanisms of Yki that have been described as phosphorylation-independent [1] or transcriptional activation mediated by Yki [40]. As mentioned above, Ser255 is also a potential target of the protein kinase ERK; therefore, the observed functional consequences of the overexpression of Ser255-mutant Yki could also be because a regulatory influence of the protein kinase ERK is lost. Future investigations should elucidate whether Yki is also a substrate of ERK, whether the localisation of Yki or its interaction with partners changes depending on its phosphorylation status and how the regulatory relationship may be constituted.

## 4. Materials and Methods 

### 4.1. Purification of Recombinant Proteins

The constructs were generated using polymerase chain reaction (PCR), performed using genomic DNA and the *yki* cDNA clone LD21311 (Berkeley *Drosophila* Genome Project) as templates. The primers came with unique restriction enzyme recognition sites added to their 5′ ends, which enabled the direct cutting of the PCR fragments. The next step was the direct cloning of the restriction-digested, sticky-end PCR fragments into the pGEX4T-1 vector and transformation into bacteria. All the constructs were verified by sequencing (Sanger). According to the manufacturer’s instructions, the GST fusion proteins were subjected to batch purification using Glutathione SepharoseTM 4B (GE Healthcare, Frankfurt, Germany). The primer combinations we used are listed in the Supplementary Material, Table S1. 

### 4.2. In Vitro Mutagenesis

To introduce Ser/Ala exchanges in the constructs, PCR mutagenesis was performed using cDNA (see above) as a template, using a mutated primer. All the constructs were sequence-checked. The resulting constructs were cloned and purified as mentioned above.

### 4.3. In Vitro Phosphorylation Assay

To analyse the phosphorylation of the recombinant proteins, in vitro kinase assays and visualisation via autoradiography were performed with recombinant GST-Hipk (~8 µg per reaction), as described previously [28]. Equal amounts of proteins (10 µg/reaction) were used for the mutational analysis. As a positive control, we used GST-tagged recombinant *Drosophila* Groucho (Gro) protein, previously identified as a substrate of Hipk [19,28]. GST served as a negative control to exclude false-positive signals from the phosphorylation of the tag. We used Hipk without any substrate to determine the background caused by the kinase’s autophosphorylation activity.

### 4.4. Fly Stocks

*Glass multiple reporter* (*GMR*)-*Gal4* (BL9146; chr.2), *scalloped* (*sd*)-*Gal4* (BL8609; chr.X), and Effector lines were generated by cloning the wildtype coding region of *yorkie* and corresponding mutant versions into the pUAST*att*B vector [41] and using phiC31 integration to insert all of the site-specific UAS constructs at the same chromosomal location (BL24871; chr.3L, 65B2; *attP*-3B) to ensure comparable expression levels for all the transgenes: UAS-*yki,* UAS-*yki*-S^169^A/S^172^A, UAS-*yki-*S^168^A/S^169^A/S^172^A and UAS-*yki-*S^255^A.

Fly strain for phiC31 integration: *y*[1] M{vas-int.Dm}ZH-2A *w*[*] PBac{*y*[+]-*attP*-3B}VK00033 (BL24871). Further effector lines used: P{UAS-*yki*.GFP}4-12-1 (BL28815; chr.2); P{UAS-*yki*.S168A.GFP.HA}10-7-1 (BL28816; chr.3); *yellow white* (*yw*^67c23^) (BL6599). The flies were reared on standard medium and raised at 25 or 18 °C as indicated.

### 4.5. Phenotypical Analysis

The fly heads were dissected from the bodies of adult female flies and mounted on coverslips. The wings were dissected from the adult female flies, rinsed in 70% EtOH and mounted on a coverslip in Aquatex^®^ mounting medium. The wings were imaged using an Olympus SZX 12 microscope with a U-CMAD3 camera system, and area measurement was performed using the cellP imaging software (Olympus, Germany). The shown images were processed with Adobe Photoshop and Adobe Illustrator.

## 5. Conclusions

In this study, we showed that the Hippo (Hpo)/Warts (Wts) signalling effector Yorkie (Yki) is an in vitro substrate of homeodomain-interacting protein kinase (Hipk) in *Drosophila melanogaster*, and we mapped several Hipk-phosphorylation sites (Ser^168^, Ser^169^/Ser^172^ and Ser^255^). In vivo functional relevance was demonstrated by expression studies with respective mutant forms of Yki. The effect of a Ser255 mutation in Yki clearly implies that Hipk can also regulate Yki in vivo via this site. Hipk is the second kinase, alongside Wts, known to phosphorylate the transcriptional coactivator Yki, implying that the regulation of Yki, in addition to the Hpo core pathway, is more complex than expected. Our results may be of general interest, as the Hpo signalling pathway is highly conserved in *Drosophila* as well as vertebrates and plays a pivotal role in maintaining homeostasis in cell proliferation and organ growth during both normal development and oncogenesis.

## Data Availability

Not applicable.

## Supplementary Materials

The supplementary materials can be found at https://www.mdpi.com/1422-0067/22/4/1862/s1.

## Abbreviations

HIPK/Hipk	Homeodomain-interacting protein kinase
Yki	Yorkie, aa amino acids

## References

[1] Badouel C., Garg A., McNeill H. (2009). Herding hippos: Regulating growth in flies and man. Curr. Opin. Cell Biol..

[2] Zheng Y., Pan D. (2019). The hippo signaling pathway in development and disease. Dev. Cell..

[3] Snigdha K., Gangwani K.S., Lapalikar G.V., Singh A., Kango-Singh M. (2019). Hippo signaling in cancer: Lessons from *Drosophila* models. Front. Cell Dev. Biol..

[4] Koontz L.M., Liu-Chittenden Y., Yin F., Zheng Y., Yu J., Huang B., Chen Q., Wu S., Pan D. (2013). The Hippo effector Yorkie controls normal tissue growth by antagonizing scalloped-mediated default repression. Dev. Cell..

[5] Yu J., Pan D. (2018). Validating upstream regulators of Yorkie activity in Hippo signaling through scalloped-based genetic epistasis. Development.

[6] Mackintosh C. (2004). Dynamic interactions between 14-3-3 proteins and phosphoproteins regulate diverse cellular processes. Biochem. J..

[7] Huang J., Wu S., Barrera J., Matthews K., Pan D. (2005). The Hippo signaling pathway coordinately regulates cell proliferation and apoptosis by inactivating Yorkie, the *Drosophila* Homolog of YAP. Cell.

[8] Dong J., Feldmann G., Huang J., Wu S., Zhang N., Comerford S.A., Gayyed M.F., Anders R.A., Maitra A., Pan D. (2007). Elucidation of a universal size-control mechanism in *Drosophila* and mammals. Cell.

[9] Hao Y., Chun A., Cheung K., Rashidi B., Yang X. (2008). Tumor suppressor LATS1 is a negative regulator of oncogene YAP. J. Biol. Chem..

[10] Zhang J., Smolen G.A., Haber D.A. (2008). Negative regulation of YAP by LATS1 underscores evolutionary conservation of the *Drosophila* Hippo pathway. Cancer Res..

[11] Zhao B., Ye X., Yu J., Li L., Li W., Li S., Yu J., Lin J.D., Wang C.Y., Chinnaiyan A.M. (2008). TEAD mediates YAP-dependent gene induction and growth control. Genes Dev..

[12] Oh H., Irvine K.D. (2008). *In vivo* regulation of Yorkie phosphorylation and localization. Development.

[13] Oh H., Irvine K.D. (2009). *In vivo* analysis of Yorkie phosphorylation sites. Oncogene.

[14] Ren F., Zhang L., Jiang J. (2010). Hippo signaling regulates Yorkie nuclear localization and activity through 14-3-3 dependent and independent mechanisms. Dev. Biol..

[15] Zhao B., Wei X., Li W., Udan R.S., Yang Q., Kim J., Xie J., Ikenoue T., Yu J., Li L. (2007). Inactivation of YAP oncoprotein by the Hippo pathway is involved in cell contact inhibition and tissue growth control. Genes Dev..

[16] Zhao B., Li L., Tumaneng K., Wang C.Y., Guan K.L. (2010). A coordinated phosphorylation by Lats and CK1 regulates YAP stability through SCF (beta-TRCP). Genes Dev..

[17] Chen J., Verheyen E.M. (2012). Homeodomain-interacting protein kinase regulates Yorkie activity to promote tissue growth. Curr. Biol..

[18] Poon C.L., Zhang X., Lin J.I., Manning S.A., Harvey K.F. (2012). Homeodomain-interacting protein kinase regulates Hippo pathway-dependent tissue growth. Curr. Biol..

[19] Kim Y.H., Choi C.Y., Lee S.-J., Conti M.A., Kim Y. (1998). Homeodomain-interacting protein kinases, a novel family of co- repressors for homeodomain transcription factors. J. Biol. Chem..

[20] Manning G., Whyte D.B., Martinez R., Hunter T., Sudarsanam S. (2002). The protein kinase complement of the human genome. Science.

[21] Link N., Chen P., Lu W.J., Pogue K., Chuong A., Mata M., Checketts J., Abrams J.M. (2007). A collective form of cell death requires homeodomain interacting protein kinase. J. Cell Biol..

[22] Choi C.Y., Kim Y.H., Kim Y.O., Park S.J., Kim E.A., Riemenschneider W., Gajewski K., Schulz R.A., Kim Y. (2005). Phosphorylation by the DHIPK2 protein kinase modulates the corepressor activity of Groucho. J. Biol. Chem..

[23] Steinmetz E.L. (2018). Analyse von Wechselwirkungen der Homeodomänen Interagierenden Proteinkinase (Hipk) mit Genen der Augenentwicklung von *Drosophila melanogaster*. Ph.D. Thesis.

[24] Rinaldo C., Prodosmo A., Siepi F., Soddu S. (2007). HIPK2: A multitalented partner for transcription factors in DNA damage response and development. Biochem. Cell Biol..

[25] Rinaldo C., Siepi F., Prodosmo A., Soddu S. (2008). HIPKs: Jack of all trades in basic nuclear activities. Biochim. Biophys. Acta Mol. Cell Res..

[26] Kuwano Y., Nishida K., Akaike Y., Kurokawa K., Nishikawa T., Masuda K., Rokutan K. (2016). Homeodomain-interacting protein kinase-2: A critical regulator of the DNA damage response and the epigenome. Int. J. Mol. Sci..

[27] Lee W., Swarup S., Chen J., Ishitani T., Verheyen E.M. (2009). Homeodomain-interacting protein kinases (Hipks) promote Wnt/Wg signaling through stabilization of β -catenin / Arm and stimulation of target gene expression. Development.

[28] Lee W., Andrews B.C., Faust M., Walldorf U., Verheyen E.M. (2009). Hipk is an essential protein that promotes Notch signal transduction in the *Drosophila* eye by inhibition of the global co-repressor Groucho. Dev. Biol..

[29] Sudol M. (2010). Newcomers to the WW domain-mediated network of the Hippo tumor suppressor pathway. Genes Cancer..

[30] Kim E.A., Noh Y.T., Ryu M.-J., Kim H.-T., Lee S.-E., Kim C.-H., Lee C., Kim Y.H., Choi C.Y. (2006). Phosphorylation and transactivation of Pax6 by homeodomain-interacting protein kinase 2. J. Biol. Chem..

[31] Dewald D.N., Steinmetz E.L., Walldorf U. (2014). Homeodomain-interacting protein kinase (Hipk) phosphorylates the small SPOC family protein Spenito. Insect Mol. Biol..

[32] Steinmetz E.L., Dewald D.N., Walldorf U. (2018). Homeodomain-interacting protein kinase phosphorylates the *Drosophila* Paired box protein 6 (Pax6) homologues Twin of eyeless and Eyeless. Insect Mol. Biol..

[33] Zhai B., Villén J., Beausoleil S.A., Mintseris J., Gygi S.P. (2008). Phosphoproteome analysis of *Drosophila* melanogaster embryos. J. Proteome Res..

[34] Gnad F., Gunawardena J., Mann M. (2011). PHOSIDA 2011: The posttranslational modification database. Nucleic Acids Res..

[35] Seger R., Krebs E.G. (1995). The MAPK signaling cascade. FASEB J..

[36] An R., Da Silva X.G., Semplici F., Vakhshouri S., Hao H.X., Rutter J., Pagano M.A., Meggio F., Pinna L.A., Rutter G.A. (2010). Pancreatic and duodenal homeobox 1 (PDX1) phosphorylation at serine-269 is HIPK2-dependent and affects PDX1 subnuclear localization. Biochem. Biophys. Res. Commun..

[37] Zhou G., Wang H., Liu S.H., Shahi K.M., Lin X., Wu J., Feng X.H., Qin J., Tan T.H., Brunicardi F.C. (2013). p38 MAP kinase interacts with and stabilizes pancreatic and duodenal homeobox-1. Curr. Mol..

[38] D’Orazi G., Cecchinelli B., Bruno T., Manni I., Higashimoto Y., Saito S., Gostissa M., Coen S., Marchetti A., Del Sal G. (2002). Homeodomain-interacting protein kinase-2 phosphorylates p53 at Ser 46 and mediates apoptosis. Nat. Cell Biol..

[39] Hofmann T.G., Möller A., Sirma H., Zentgraf H., Taya Y., Dröge W., Will H., Schmitz M.L. (2002). Regulation of p53 activity by its interaction with homeodomain- interacting protein kinase-2. Nat. Cell Biol..

[40] Oh H., Slattery M., Ma L., White K.P., Mann R.S., Irvine K.D. (2014). Yorkie promotes transcription by recruiting a histone methyltransferase complex. Cell Rep..

[41] Bischof J., Maeda R.K., Hediger M., Karch F., Basler K. (2007). An optimized transgenesis system for *Drosophila* using germ-line-specific phiC31 integrases. Proc. Natl. Acad. Sci. USA.

