# Supplementary material for: Drosophila Homeodomain-Interacting Protein Kinase (Hipk) Phosphorylates the Hippo/Warts Signalling Effector Yorkie
Source: Int J Mol Sci. 2021 Feb 13;22(4):1862. doi: 10.3390/ijms22041862 (PMC7918113; doi:10.3390/ijms22041862)
Supplement: Supplementary file 1 [file ijms-22-01862-s001.zip › ijms-1077902_supplementary figure A1.docx]

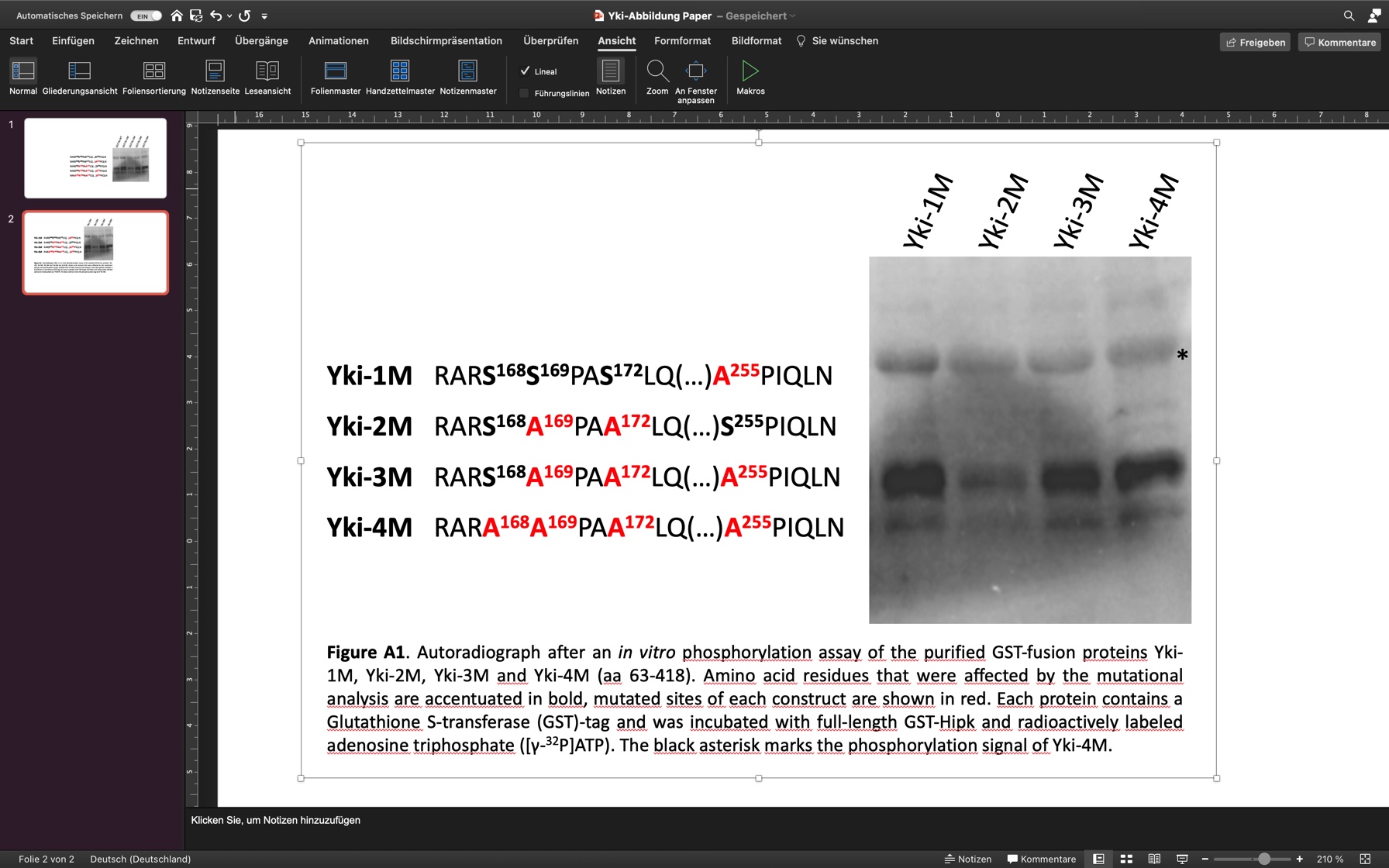


**Figure A1**. Autoradiograph after an *in vitro* phosphorylation assay of the purified GST-fusion proteins Yki-1M, Yki-2M, Yki-3M and Yki-4M (aa 63-418). Amino acid residues that were affected by the mutational analysis are accentuated in bold, mutated sites of each construct are shown in red. Each protein contains a Glutathione S-transferase (GST)-tag and was incubated with full-length GST-Hipk and radioactively labeled adenosine triphosphate ([γ-^32^P]ATP). The black asterisk marks the phosphorylation signal of Yki-4M.

We tested if the mutation of S168A (in addition to mutated S169A, S172A and S255A) influences the phosphorylation signal that was observed with Yki-3M. Again, the construct Yki-4M was tested in the shortened form (aa 63-418). In contrast to our observation with the shortened fragments (see Figure 1 C, D: fragments Yki-N2G and Yki-N2FG), the unchanged phosphorylation signal in Yki-4M implies that Ser168 might not be a main phosphorylation site of Hipk. Therefore, the remaining phosphorylation signal in Yki-4M might be within the region aa 94-118 (construct Yki-D, Figure 1).
